# Cholesteryl Ester Transfer Protein (CETP) expression does not affect glucose homeostasis and insulin secretion: studies in human CETP transgenic mice

**DOI:** 10.1186/s12944-016-0179-6

**Published:** 2016-01-13

**Authors:** Helena F. Raposo, Emerielle C. Vanzela, Jairo A. Berti, Helena C F Oliveira

**Affiliations:** Department of Structural and Functional Biology, Institute of Biology, State University of Campinas, Unicamp - Cidade Universitária Zeferino Vaz. Rua Monteiro Lobato, 255, Campinas, SP CEP 13083-862 Brazil; Present address: Department of Physiological Science, State University of Maringa, Maringa, PR Brazil

**Keywords:** Cholesteryl ester transfer protein (CETP), Glucose homeostasis, Insulin sensitivity, Glucose uptake

## Abstract

**Background:**

Cholesteryl ester transfer protein (CETP) is a plasma protein that mediates the exchange of triglycerides for esterified cholesterol between HDL and apoB-lipoproteins. Previous studies suggest that CETP may modify glucose metabolism in patients or cultured cells. In this study, we tested if stable CETP expression would impair glucose metabolism.

**Methods:**

We used human CETP transgenic mice and non-transgenic littermate controls (NTg), fed with control or high fat diet, as well as in dyslipidemic background and aging conditions. Assays included glucose and insulin tolerance tests, isolated islets insulin secretion, tissue glucose uptake and adipose tissue GLUT mRNA expression.

**Results:**

CETP expression did not modify glucose or insulin tolerance in all tested conditions such as chow and high fat diet, adult and aged mice, normo and dyslipidemic backgrounds. Fasting and fed state plasma levels of insulin were not differ in CETP and NTg mice. Direct measurements of isolated pancreatic islet insulin secretion rates induced by glucose (11, 16.7 or 22 mM), KCl (40 mM), and leucine (10 mM) were similar in NTg and CETP mice, indicating that CETP expression did not affect β-cell function *in vivo* and *ex vivo*. Glucose uptake by insulin target tissues, measured *in vivo* using ^3^H-2-deoxyglucose, showed that CETP expression had no effect on the glucose uptake in liver, muscle, perigonadal, perirenal, subcutaneous and brown adipose tissues. Accordingly, GLUT1 and GLUT4 mRNA in adipose tissue were not affected by CETP.

**Conclusions:**

In summary, by comparing the *in vivo* all-or-nothing CETP expressing mouse models, we demonstrated that CETP per se has no impact on the glucose tolerance and tissue uptake, global insulin sensitivity and beta cell insulin secretion rates.

## Background

Type 2 Diabetes mellitus (T2DM) is a metabolic disorder characterized by high glucose plasma levels and insulin resistance [1]. Insulin resistant diabetic patients commonly present dyslipidemia, which is generally characterized by high triglycerides and low HDL plasma levels [2]. The HDL-cholesterol level is recognized as a protective factor against atherosclerosis and cardiovascular diseases because HDL promotes reverse cholesterol transport from the periphery to the liver and acts as anti-inflammatory, antioxidant and anti-thrombotic lipoprotein [3].

Cholesteryl ester transfer protein (CETP) is a plasma protein that mediates the exchange of triglycerides from apoB-lipoproteins for esterified cholesterol from HDL [4]. In this way, CETP promotes reduction of plasma HDL-cholesterol and, thus, may increase the risk of atherosclerosis. Therefore, in the last decades, efforts have been made to inhibit CETP activity as an anti-atherogenic intervention. Although these CETP inhibitors increase HDL-cholesterol and decrease LDL-cholesterol concentrations, they failed to prove their benefits in reducing cardiovascular disease mortality [5–7].

There are evidences that suggest a relationship between CETP and glucose homeostasis, although this issue has been a matter of debate. Clinical studies associated higher CETP levels to T2DM risk [8] and showed that CETP polymorphism may modulate insulin sensitivity [9, 10]. Individuals with higher CETP mass present higher values of fasting insulin and HOMA insulin resistance index compared with individuals with lower CETP mass. A post-hoc analysis of the ILLUMINATE trial linked CETP inhibition by torcetrapib to a significant improvement in glycemic control [11].

On the other hand, comparison of subject groups with distinct insulin sensitivity (lean *vs.* obese and rested *vs.* exercised) found no association between CETP and insulin sensitivity [12]. In addition, through a genome-wide approach, Sladek et al. [13] performed a systematic search looking for associations between T2DM risk and almost 400,000 polymorphisms. They did not find any significant associations between major genes or proteins associated with HDL metabolism and risk of T2DM or glucose intolerance. Furthermore, a recent clinical trial using a potent CETP inhibitor, dalcetrapib, failed to improve insulin sensitivity in patients [14].

In the opposite direction, Ju et al. [15] found that expressing human CETP in 3T3-L1 cells resulted in increased glucose uptake, suggesting that CETP expression would improve insulin sensitivity. Accordingly, Cappel et al. [16] reported that simian CETP expression protects against insulin resistance in obese female mice.

In light of these conflicting evidences of the role of CETP on the glucose and insulin homeostasis, we took the advantage of the human CETP transgenic mice model, expressing physiological levels of CETP, and compared them with their CETP non-expressing littermate controls to further investigate this issue.

## Results

Human CETP transgenic mice were used to verify the effects of CETP long-term expression over glucose metabolism. Heterozygous CETP transgenic (CETP-Tg) mice expressing a human natural promoter-driven CETP transgene [17] were compared to their non-transgenic littermates mice. These CETP transgenic mice present human like plasma levels of CETP (~2 ug/ml) [18], modest reductions of HDL-cholesterol plasma levels [19], authentic tissue pattern of expression and regulation of gene expression [17, 20].

**Fig. 1:**
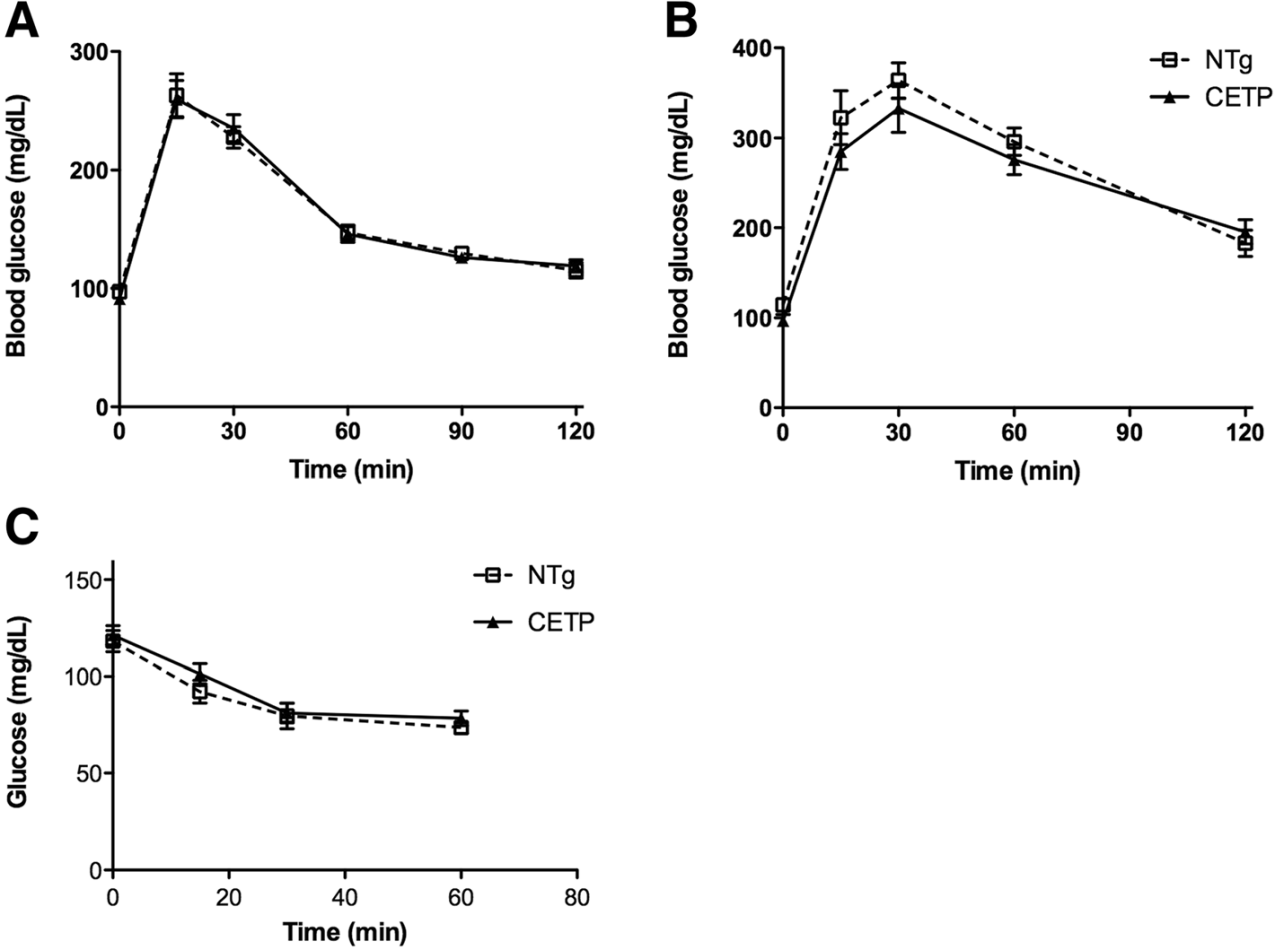
CETP expression does not affect either glucose tolerance or insulin sensitivity. Glucose tolerance test (GTT) was performed in 5–9 month old NTg and CETP female (**a**: oral GTT) (n = 8 each group) and male (**b**: ip GTT) (n = 5 each group) mice, fasted for 12 h. lntraperitoneal (*ip*) insulin-tolerance test (ITT) was performed in fed mice (**c**) (NTg: n = 2 male and 2 female, CETP: n = 2 male and n = 3 female). Values are expressed as mean ± SE. Student’s *t* test: non-significant

**Fig. 2:**
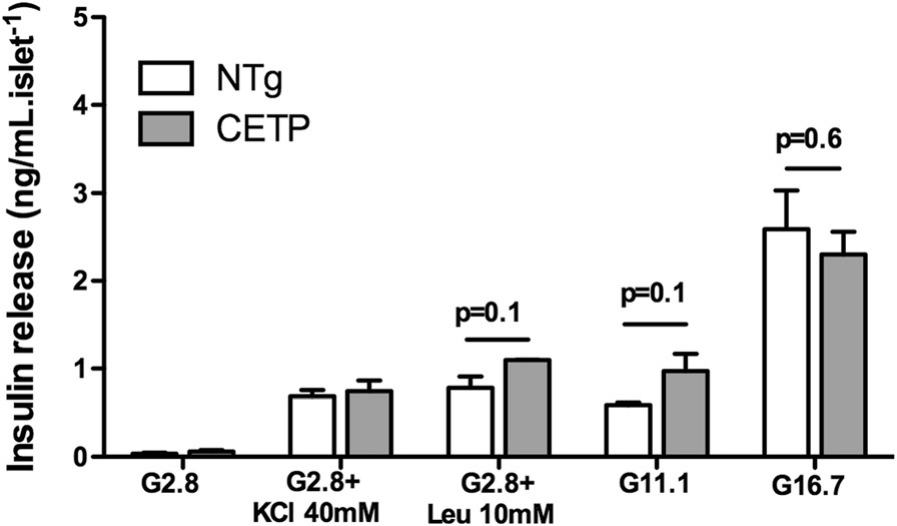
CETP expression does not impact on insulin secretion. Freshly isolated islets from female NTg (white columns) or CETP (gray columns) mice with 7 month of age were incubated in Krebs bicarbonate buffer containing 5.6 mmol/l glucose for 30 min at 37 °C (5 islets/well, four replicates in each condition). Then, the buffer was replaced by fresh buffer containing 2.8 mM glucose, 2.8 mM glucose plus KCl (40 mM), 2.8 mM glucose plus leucine (10 mM), 11 mM glucose or 16.7 mM glucose. After 1 h incubation, buffer was collected and insulin was measured by radioimmunoassay. Insulin release was normalized by islet number. Values are expressed as mean ± S.E. n = 4–6 mice in 3 independent experiments. Student’s t test: non-significant

As shown in Fig. 1, the glycemic curves after a glucose load (GTT) in female (Fig. 1a) and male (Fig. 1b) mice and after an insulin load (ITT) were similar between CETP and NTg mice. Therefore, CETP expression apparently does not modify either glucose tolerance or insulin sensitivity. To confirm these results, we also measured plasma insulin levels in both fasting and fed states in male and female mice (Table 1) and found no effects of CETP expression on the insulinemia. Next we evaluated the *ex vivo* insulin secretion by isolated pancreatic islets stimulated by several secretagogues (Fig. 2). Glucose (11 mM or 16.7 mM), KCl (40 mM), and leucine (10 mM) induced insulin secretion in a similar manner in islets isolated from NTg or CETP mice, indicating that CETP expression does not affect β-cell function.

**Table 1:** CETP expression has no effect on insulin plasma levels

		Plasma insulin (ng/ml)	
		NTg	CETP
Female	Fast	0.330 ± 0.023 (9)	0.380 ± 0.040 (5)
	Fed	0.488 ± 0.082 (9)	0.970 ± 0.604 (5)
Male	Fast	0.240 ± 0.108 (3)	0.447 ± 0.047 (4)
	Fed	0.464 ± 0.024 (3)	0.619 ± 0.066 (4)

Insulin plasma level of male and female CETP expressing mice and their littermates’ controls in fed state and after 12 h fast. Values are expressed as mean ± SE (n). Student’s *t* test: NTg *vs.* CETP: non-significant

**Fig. 3:**
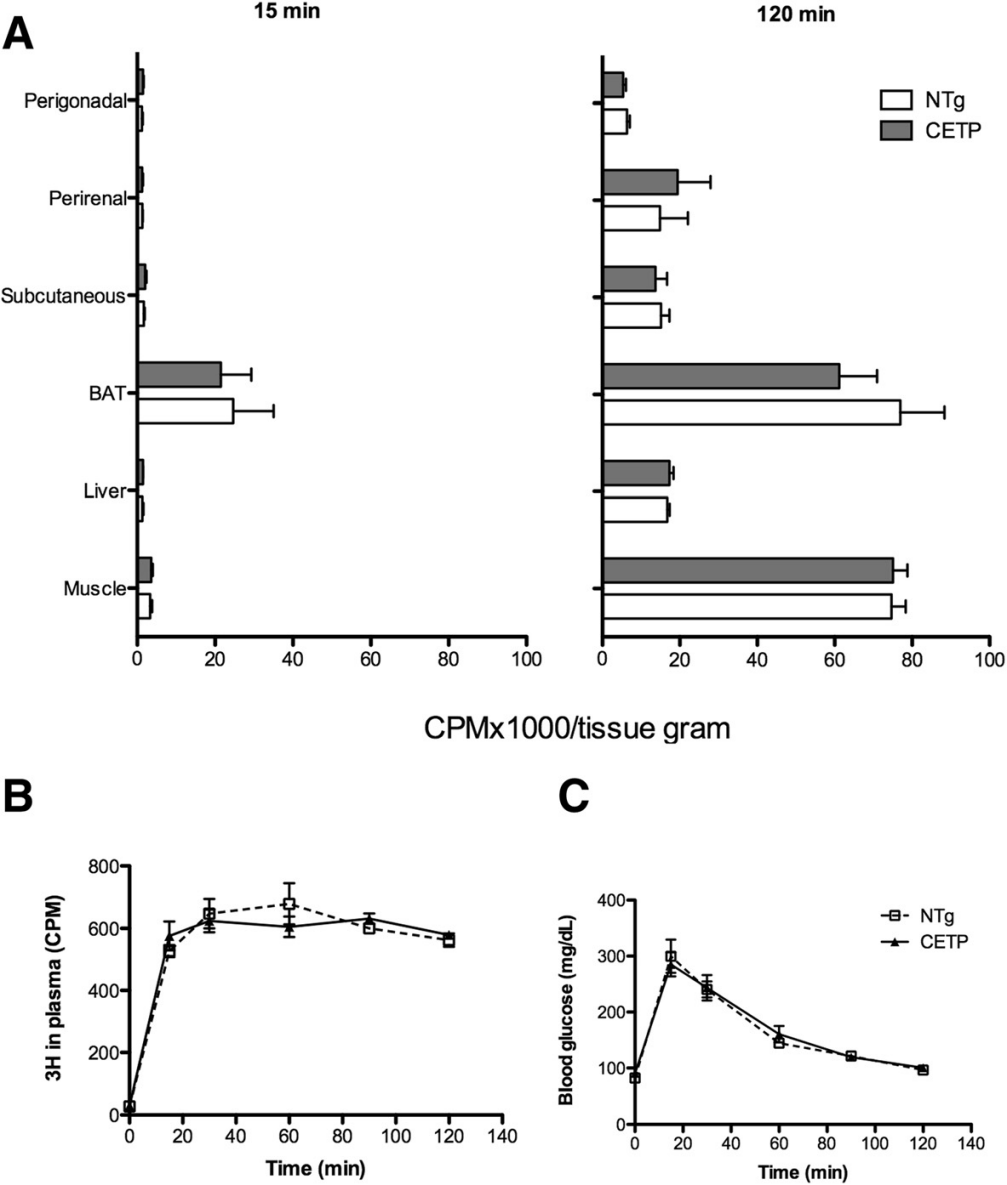
CETP expression has no effect on tissue glucose uptake. Perigonadal, perirenal, subcutanous and brown (BAT) adipose tissue, liver and muscle glucose uptake was measured i*n vivo*, 15 min and 2 h after an intraperitoneal injection of glucose (2 g/Kg BW) and ^3^H-2-deoxiglucose (150 μCi/Kg BW) in 5 month old NTg and CETP female mice (**a**). Blood was collected ^3^H activity was determined in plasma (**b**) and glucose (**c**) in blood. Mean ± SE (n = 3–6 per group). Student’s *t* test: non-significant

**Fig. 4:**
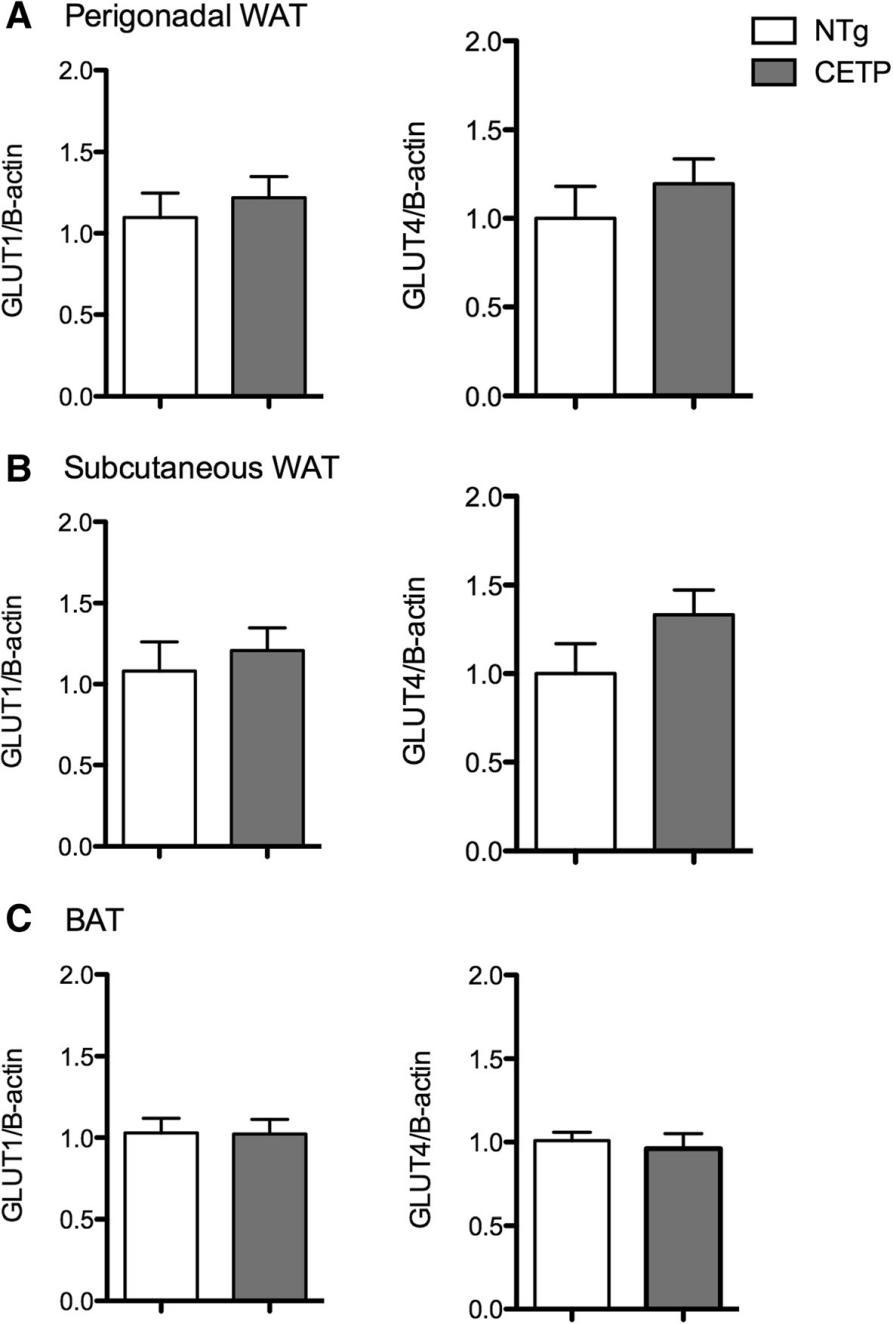
CETP expression does not affect glucose transporters gene expression in adipose tissues. *Scl2a1* (encoding GLUT1) and *Scl2a1* (encoding GLUT4) mRNA expression were determined in visceral (**a**), subcutaneous (**b**) and brown (**c**) adipose tissue depots of 5 months old non-transgenic (NTg) and CETP female mice. Gene expression was quantified by real-time RT-PCR using the ΔΔCT method normalized to β-actin and then expressed relative to the control (NTg) group. Mean ± SE (n = 7–12 per group). Student’s *t* test: non-significant

Tissue specific sensitivity to insulin was directly determined *in vivo* by measuring glucose uptake by white (WAT) and brown (BAT) adipose tissues, liver and muscle after a bolus *ip* injection of ^3^H-2-deoxiglucose (^3^H-2DG) dissolved in a glucose solution (2 g/Kg BW). As shown in Fig. 3, CETP expression had no effects on the glucose tissue uptake when compared to NTg mice, as well as it did not modify the plasma clearance of ^3^H-2DG and glucose levels. Since a previous study [15] reported that 3T3 mouse adipocytes transfected with CETP cDNA presented increased glucose uptake, we checked the expression of genes encoding the glucose transporters GLUT1 (*Scl2a1*) and GLUT4 (*Scl2a4*) mRNA levels in the mice adipose tissue (Fig. 4). In agreement with the results of *in vivo* glucose uptake, CETP expression did not change GLUT gene expression in the adipose tissue depots.

**Fig. 5:**
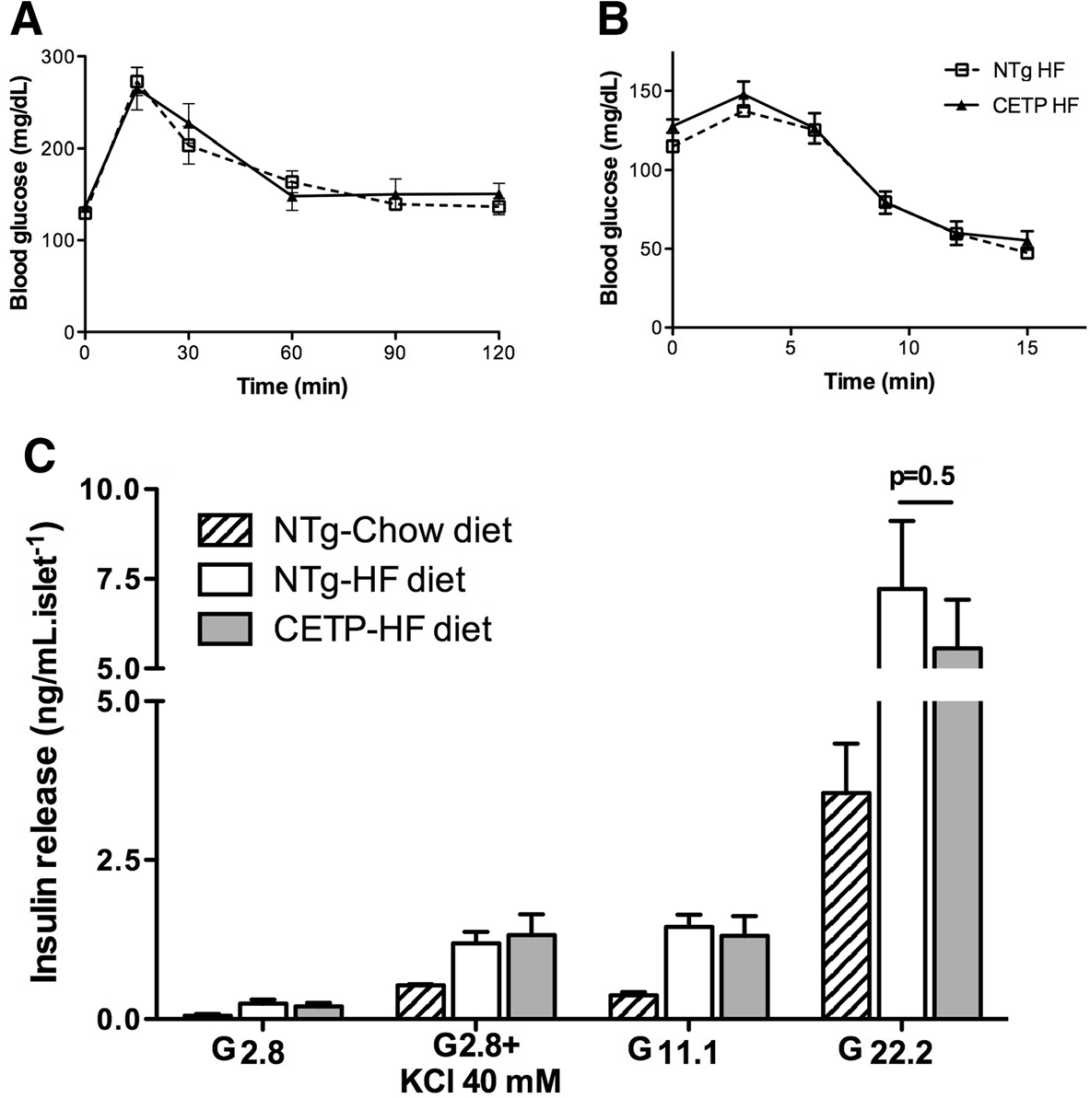
CETP expression does not affect glucose tolerance, insulin sensitivity and secretion in obese mice. Glucose tolerance test performed in NTg and CETP female mice treated with high fat diet from 2 to 5 months of age, after 12 hs of fasting, n = 5–7 per group (**a**). lntraperitoneal (ip) insulin-tolerance test (ITT) was performed after 3 h fasting, n = 6–8 (**b**). Freshly isolated islets from NTg mice fed with chow diet (black columns), and NTg (white columns) and CETP (gray columns) mice fed with high fat diet were incubated in the presence of 2.8 mM glucose, 2.8 mM glucose plus KCl (40 mM), 11 mM glucose or 22.2 mM glucose, as described in Fig. 2, n = 2–4 female mice in two independent experiments (**c**). Values are expressed as mean ± S.E. Student’s *t* test: non-significant

To further investigate if CETP would affect glucose metabolism in the presence of other metabolic complications, glucose tolerance tests were performed in aged or dyslipidemic backgrounds. For this purpose, we crossbred CETP mice with a model of hypertriglyceridemia due to overexpression of apolipoprotein CIII (CETP^+/−^/CIII^−/+^) and with a model of hypercholesterolemia due to the disruption of the LDL receptor gene (CETP^+/−^/LDLr^−/+^). These crosses were previously studied and exhibit marked hypertriglyceridemia and mild hypercholesterolemia, respectively [21, 22]. Table 2 shows that CETP expression had no effect on the glucose tolerance in male aged (18 month old), hypertriglyceridemic (apoCIII overexpressing) or LDL receptor defective mice. In addition, possible effects of CETP on glucose homeostasis were also investigated in the context of a high fat diet (60 % Kcal as saturated fat) for 3 months. As shown in Fig. 5, CETP expression did not affect glucose tolerance (Fig. 5a), insulin sensitivity (Fig. 5b) and islet insulin secretion (Fig. 5c) in high fat fed mice. As expected, insulin secretion was higher in all mice fed with a high fat diet compared to chow diet. However, the comparison between NTg and CETP mice showed no significant differences in insulin secretion (Fig. 5c).

**Table 2:** CETP expression has no effect on glucose tolerance in aged, hypertriglyceridemic or hypercholesterolemic mice

Model	AUC ± SE	n
	(mg.dl^−1^.min)	male/female
Aged-NTg	10933 ± 1570 (4)	4/0
Aged-CETP	11737 ± 1069 (7)	7/0
ApoCIII^−/+^	22777 ± 3238 (4)	3/1
ApoCIII^−/+^ /CETP^−/+^	25114 ± 9466 (3)	1/2
LDLr^−/+^	15920 ± 2587 (4)	0/4
LDLr^−/+^/CETP^+/−^	12951 ± 407 (5)	2/3

Area under the curve (AUC) of oral glucose tolerance test in aged (18 month old), apo CIII transgenic and LDL receptor deficient (LDLr^−/+^) mice expressing or not CETP, fasted for 12 h. At time zero, mice received a glucose dose of 1.5 g/kg body weight by gavage. Samples for glucose determination were collected from the tail tip at 0, 15, 30, 60 and 120 min. Values are expressed as mean ± SE (n: male and female pooled data). Student’s *t* test for all comparisons: non-significant

## Discussion

Several previous studies have suggested a relationship between CETP and glucose metabolism [8–10, 15, 16] including a putative positive effect of pharmacological inhibition of CETP on the glycemic control [11, 23]. Thus, in this study we used mice expressing a human CETP gene to investigate the long-term consequences of CETP on the glucose homeostasis. Because wild type mice do not express this gene [24], CETP transgenic mice are useful to explore CETP effects in a complex in vivo biological system. We investigated the main steps of glucose homeostasis and verified that CETP expression causes no significant alterations in glucose tolerance, fasting and fed plasma insulin levels, global insulin sensitivity, pancreatic islet insulin secretion and glucose tissue uptake. Previous data from our group had already shown no differences in the plasma levels of glucose and insulin, either in fasted or fed state, in CETP transgenic compared to NTg mice [25]. Therefore, according to our data, the CETP expression does not impose any disadvantage for glucose homeostasis maintenance.

Ju et al. have previously shown that *in vitro* human CETP expression in adipocyte 3T3-L1 mouse cell line (driven by a heterologous viral promoter) resulted in enhanced insulin-mediated glucose uptake [15]. Here, we demonstrate that *in vivo* human CETP expression driven by its natural promoter did not affect glucose uptake by adipose and other insulin target tissues. It is likely that the *in vivo* hormonal milieu present in the whole organism are responsible for these distinct results.

Briand et al. [26] demonstrated in hamsters that CETP inhibition by torcetrapib treatment significantly reduced blood glucose and plasma insulin levels after a high fat/high cholesterol diet. ^3^H-2DG uptake was reduced in adipose tissues but increased in soleus, liver and heart of these torcetrapib treated hamsters. However, the pattern of CETP tissue expression in hamsters markedly differs from that observed in the transgenic mice, which is similar to humans [17, 27]. Contrary to the effect observed in hamsters, torcetrapib treatment of humans induced an increase in post-prandial plasma insulin levels [23].

Cappel et al. [16] used a mouse model expressing a simian CETP gene under control of a constitutive promoter to demonstrate that CETP expression protects against diet induced insulin resistance in obese female mice, but not in male or in chow fed mice. They postulate that CETP would increase cholesterol delivery to the liver, increasing bile acids excretion into the gut. These effects would initiate signaling pathways that improve insulin sensitivity, such as those observed after an oral taurodeoxycholic acid treatment [28]. Nevertheless, the CETP transgenic mice used here present no alterations in bile acids secretion into the bile and fecal excretion [29]. In addition, bile acids mass excretion is not altered in torcetrapib-treated hamsters [26].

Siebel et al. [23] showed that plasma post-CETP inhibition promoted glucose-stimulated insulin secretion in MIN6N8 insulinoma cells that were pretreated with oxidized (ox) LDL. The authors attributed these effects to the higher concentrations of HDL-cholesterol in the incubation medium rather than to the low CETP levels. In fact, it was previously shown that oxLDL exerts deleterious effects on beta cells, such as activation of JNK pathway and apoptosis, which are countered by HDL particles [30]. One way by which high HDL-cholesterol levels would improve the efficiency of insulin secretion could be through removing cholesterol from the cell membrane. As previously demonstrated by our group, both abnormally high or low islet cholesterol content impairs glucose stimulated insulin secretion, while its normalization improves it [31, 32]. It is important to emphasize that CETP expression decreases HDL-cholesterol levels in a gene dosage manner [19]. The mouse model used here exhibits about 15–20 % lower HDL-cholesterol levels when compared with NTg mice [19, 22]. Even though, here we verified no differences in insulin secretion of freshly isolated islets from NTg or CETP mice.

Lopez-Rıos et al. [10] have hypothesized that individuals with higher CETP activity have HDL particles enriched in triglycerides, which would increase the flux of free fatty acid to the liver and decrease hepatic sensitivity to insulin, and thus, predispose to diabetes. According to De Vries et al. [8], CETP mass and activity was higher in T2DM subjects, suggesting that lowering plasma CETP could ameliorate diabetes-associated cardiovascular risk. Alongside, Barter et al. [11] reported that treatment with torcetrapib improves glycemic control in atorvastatin-treated patients with T2DM. Since literature shows that CETP have worse outcomes in unhealthy patients, here CETP effects were also investigated in mice treated with high fat diet, with hyperlipidemic background, and also in aged mice. However, our data show no harm of CETP stable expression even when metabolism was challenged to more adverse conditions. Since different results were found with CETP expressing mice and with CETP inhibitor torcetrapib [11, 23], it is also possible to speculate about a direct effect of this drug on the glycemic control, independently of the CETP levels. Our data is in agreement with previous work that showed that CETP activity does not correlate with parameters of insulin sensitivity in non-diabetic individuals [12]. A conciliatory view of most conflicting clinical and experimental data is that CETP per se is not relevant to glucose homeostasis/insulin sensitivity, but actually the HDL-cholesterol levels and perhaps the HDL functionality is important to regulate glucose metabolism and modulate diabetes risk [33]. As mentioned before, the CETP mouse model studied here present modest changes in HDL plasma levels.

## Conclusions

In conclusion, by comparing *in vivo* all-or-nothing human CETP mouse model, our data demonstrate that CETP *per se* does not modulate glycemic control and insulin secretion in young and aged healthy mice, as well as in dyslipidemic and high fat fed mice.

## Methods

### Animals

All experimental protocols were approved by the university’s Committee for Ethics in Animal Experimentation (CEUA/UNICAMP, protocol # 1107-1) and are in accordance with the “Principles of Laboratory Animal Care” (NIH publication no. 85–23, revised 1985). In this study, experiments were performed in 5 to 9 months old mice (adult), and in an additional aged group of 18 months old mice. Both male and female mice were used for the experiments. Heterozygous CETP transgenic (CETP-Tg) mice expressing a human natural promoter-driven CETP transgene, line 5203 [17] and non-transgenic littermates mice were used. CETP-Tg mice were crossbred with human apolipoprotein CIII transgenic mice, line 3707 [34] to generate apoCIII and apoCIII/CETP. In addition, LDL receptor (LDLr^−/−^) knockout mice [35] and CETP-Tg mice were crossbred to generate LDLr^−/+^ and CETP^+/−^/ LDLr^−/+^ mice. CETP-expressing mice were screened by assaying plasma CETP activity, as previously described [18]. The apoCIII transgenic mice had plasma triacylglycerol levels above 300 mg/dL and LDLr^−/−^ mice had plasma cholesterol levels above 200 mg/dL, whereas the non-transgenic mice had both triacylglycerol and cholesterol levels below 100 mg/dL. Mice were housed in a temperature-controlled room (22 ± 1 °C) on a 12 h light/dark cycle and had free access to food and water. The chow diet (Nuvital CR1, Colombo, Brazil) was offered from weaning and its composition was (% by weight) 23 % protein, 4.5 % total fat, 33 % carbohydrates and 21 % fiber, with a total of 263 kcal/100 g. The high fat diet was offered from 2 to 5 months old and its composition was (% by weight) 14 % protein, 35 % total fat (59 % of calories), 41 % carbohydrates and 5 % fiber, with a total of 536 kcal/100 g.

### Blood glucose and plasma insulin levels

Blood glucose concentrations were measured using a glucose analyzer (Accu-Chek Advantage, Roche Diagnostic, Switzerland). Plasma insulin levels were determined by ELISA (Ultra Sensitive Mouse Insulin ELISA Kit, Christal Chem, USA) according to the manufacturer instructions.

### Glucose tolerance test (GTT) and insulin tolerance test (ITT)

Female mice were submitted to an oral glucose tolerance test after 12 h fast. Basal blood sample was collected from the tail tip (t = 0 min) and a glucose load of 1.5 g/kg body weight was then administered by oral gavage. Additional blood samples were collected at 15, 30, 60 and 90 min after glucose ingestion. Male mice were submitted to an intraperitoneal (*ip*) glucose tolerance test. After 12 h fast, mice were injected with a glucose load of 2 g/kg body weight and blood samples were collected at 0, 15, 30, 60 and 120 min. For the ITT, blood was taken from fed female and male mice immediately before insulin *ip* injection [0.75 U/Kg body weight of human insulin (Human insulin R, Eli Lilly and Company)] and at the times 10, 15, 30 and 60 min for glucose analyses.

### Pancreatic islet isolation and insulin secretion

The pancreatic islets were isolated from fed mice by collagenase digestion (0.8 mg/mL; Type V; Sigma C9263) (adapted from [36]), rinsed tree times in Hanks Solution [137 mmol/l NaCl; 5.4 mmol/l KCl; 0.8 mmol/l MgSO_4_.7H_2_0; 0.34 mmol/l Na_2_HPO_4_; 0,44 mmol/l KH_2_PO_4_; 1.26 mmol/l CaCl_2_.2H_2_0; 4.2 mmol/l NaHCO_3_; pH 7.4 plus 5.6 mmol/l glucose and 0.1 % (w/v) BSA] to remove the enzyme from medium and then selected manually under a microscope to exclude any contaminating tissues. After isolation, batches of 5 islets each were pre-incubated in Krebs-Ringer bicarbonate buffer (KRBB) containing: 115 mmol/l NaCl, 5 mmol/l KCl, 10 mmol/l NaHCO_3_, 2.5 mmol/l CaCl_2_.2H_2_O, 1 mmol/l MgCl_2_.6H_2_O and 15 mmol/l HEPES, pH 7.4 plus 5.6 mmol/l glucose and 0.3 % (w/v) BSA for 30 min at 37 °C. Then, the buffer was replaced by fresh buffer containing 2.8 mM glucose, 2.8 mM glucose plus KCl (40 mM), 2.8 mM glucose plus leucine (10 mM), 11 mM glucose, 16.7 mM glucose or 22.2 mM glucose. After 1 h incubation, buffer was collected and insulin was measured by radioimmunoassay [37] using rat insulin as standard, human recombinant ^125^I-Insulin (PerkinElmer) and anti-insulin from guinea pig. Insulin release was normalized by islet number.

### Glucose tissue uptake (*in vivo*)

After 12 h fasting, mice were injected (i.p.) with glucose (2 g/Kg body weight) plus ^3^H-2-deoxiglucose (150 μCi/Kg body weight) [38]. Blood sample were collected from the tail tip before (time 0) and after the injection at 15, 30, 60, 90 e 120 min for glucose and radioactivity determination. At 15 min and 120 min, groups of mice were anesthetized and killed by exsanguination. Tissues [liver, muscle (gastrocnemius + soleus), perigonadal, perirenal, subcutaneous and brown adipose tissue] were collected, washed with saline, and digested in a tissue solubilizer solution (GE Healthcare-Amersham). Non-aqueous cintilation liquid (GE Healthcare-Amersham) were added to count ^3^H radioactivity in the samples using a beta-counter (Bekmam - LS 6000TA). The quench caused by sample color was corrected by adding 1000 cpm of ^3^H-triolein, providing proportional correction.

### RNA extraction and real-time RT-PCR

Total RNA from adipose tissue depots were purified using RNeasy Lipid Tissue Mini Kit (QIAGEN, Germany), according to the manufacture’s protocol. The integrity of the RNA was assessed using Tris-borate 1.2 % agarose gels stained with ethidium bromide. The amount and purity of the RNA were determined by optical density readings at 260 and 280 nm (Gene Quant, Amersham-Pharmacia Biotech). Genomic DNA contamination was excluded by running a PCR on the RNA samples. cDNA was obtained from 2 μg of total RNA by reverse transcription using an Applied Biosystems kit (High-Capacity cDNA reverse transcription kit) according to the manufacturer’s instructions. mRNA expression was determined by real-time RT-PCR (Step One Real-time PCR System, Applied Biosystems, Foster City, CA) using a SybrGreen PCR Master Mix and the specific primers (Table 3).

**Table 3:** Specific primers sequences used for Real-time PCR

Gene	Primers	
β-actin	forward	5′ GGACTCATCGTACTCCTGCTT 3′
	reverse	5′ GAGATTACTGCTCTGGCTCCT 3′
*Scl2a1*	forward	5′CCATCCACCACACTCACCACGC-3′
	reverse	5′GCCCATAAGCACAGCAGCCACA-3′
*Scl2a4*	forward	5′-ACATACCTGACAGGGCAAGG-3′
	reverse	5′-CGCCCTTAGTTGGTCAGAAG-3′

Gene expression was quantified using the ΔΔCT method by measuring the threshold cycle normalized to β-actin and then expressed relative to the control groups.

## Abbreviations

T2DM: Type 2 Diabetes mellitus
CETP: Cholesteryl ester transfer protein, Apo, apolipoproteins
oxLDL: Oxidized LDL
